# Adverse Reactions to the Orphan Drug Cerliponase Alfa in the Treatment of Neurolipofuscinosis Type 2 (CLN2)

**DOI:** 10.3390/ph17111513

**Published:** 2024-11-11

**Authors:** Ilaria Ammendolia, Maria Sframeli, Emanuela Esposito, Luigi Cardia, Alberto Noto, Mariaconcetta Currò, Gioacchino Calapai, Maria De Pasquale, Carmen Mannucci, Fabrizio Calapai

**Affiliations:** 1 Department of Clinical and Experimental Medicine, University of Messina, 98125 Messina, Italy; ilaria.ammendolia@unime.it (I.A.); maria.sframeli@polime.it (M.S.); mariaconcetta.curro@unime.it (M.C.); fabrizio.calapai@unime.it (F.C.); 2 Department of Chemical, Biological, Pharmaceutical and Environmental Sciences, University of Messina, 98125 Messina, Italy; emanuela.esposito@unime.it (E.E.); gioacchino.calapai@unime.it (G.C.); carmen.mannucci@unime.it (C.M.); 3 Department of Human Pathology of Adult and Childhood “Gaetano Barresi”, University of Messina, 98125 Messina, Italy; alberto.noto@unime.it (A.N.); maria.depasquale@unime.it (M.D.P.); 4 Department of Biomedical and Dental Sciences and Morphological and Functional Imaging, University of Messina, 98125 Messina, Italy

**Keywords:** cerliponase alfa, pharmacovigilance, adverse reactions, neurolipofuscinosis, EudraVigilance, pleocytosis

## Abstract

**Background/Objectives:** Neuronal Ceroid Lipofuscinosis type 2 is a rare pathology affecting mainly the central nervous system (CNS) and retina, and is caused by variants in the gene encoding the lysosomal enzyme tripeptidyl peptidase 1. Therapy with enzyme replacement through the brain infusion of the orphan drug cerliponase alfa, a recombinant human tripeptidyl peptidase 1 enzyme replacement therapy delivered via intracerebroventricular infusion, has been approved for Neuronal Ceroid Lipofuscinosis type 2 disease. The safety profile of cerliponase alfa has been established based on pre-authorization studies; currently, no post-marketing investigation has been performed to confirm it. Here, a descriptive analysis of real-world spontaneous reporting data of suspected adverse reactions (SARs) to cerliponase alfa in the EudraVigilance database was performed to compile clear information on the safety profile. **Methods:** Suspected adverse reactions to cerliponase alfa reported in the data system EudraVigilance were analyzed for age, sex of the patient, adverse reactions, and the indication for use. **Results**: Cases with suspected adverse reactions to cerliponase alfa were found to be more frequent in female patients (58.1%) and in children aged 3–11 years. The most common adverse reactions were, in decreasing order, fever/pyrexia, device-related infection, vomiting, seizures/convulsions, pleocytosis, irritability, ventriculitis, and respiratory disorders. **Conclusions:** The results confirm the safety profile of cerliponase alfa established with pre-registration clinical studies but suggest the need for further studies to investigate the occurrence of adverse reactions, as possible predictive prognostic markers, in more depth.

## 1. Introduction

Rare diseases are pathologies showing a low incidence, while orphan drugs are substances intended and authorized for the treatment of rare diseases. Orphan drugs were initially defined as a therapy whose costs exceeded the potential gain, but subsequently all drugs for any rare disease were included [1]. About 6000–8000 rare diseases are known in the world, with approximately 80% of them being hereditary [2]. Neuronal Ceroid Lipofuscinosis is a rare pathology represented by a heterogeneous group of 13 neurodegenerative diseases mainly affecting the central nervous system (CNS) and retinas, in which the ceroid lipofuscin is stored in lysosomes. These diseases are named with the acronym CLN and a number referring to the recessive mutation of a gene. They are a group of lysosomal storage disorders deriving from genetic mutations which are traditionally clustered based on shared clinical symptoms. According to the genetic mutation, these disorders can sometimes be identified in adults, though they are commonly referred to as pediatric neurodegenerative diseases [3]. CLNs are classified into different types based on their intrinsic origin such as lysosomal enzyme deficiencies or non-enzymatic deficiencies [4]. To date, 13 NCLs have been reported (CLN1–CLN8, CLN10–CLN14), produced by mutations in different genes [5,6,7]. They are rare, even though they are the most common neurodegenerative pathologies during infancy and childhood. Seizures, motor alterations (such as movement disorders and muscle spasms), cognitive decline, blindness, cerebellar atrophy, and behavioral problems are the major clinical features. The incidence varies between 0.6 and 14/100,000 newborns in different countries [8].

CLN2 is an autosomal recessive pathology caused by mutations in the CLN2/tripeptidyl-peptidase 1 (TPP1) gene located on chromosome 11p15.4, which encodes the TPP1 enzyme. The TPP1 enzyme is a proenzyme, which, in turn, is processed in lysosomes into the active enzyme that cleaves proteins into tripeptides during protein degradation. Mutations in the CLN2/TPP1 gene result in impaired activity of the enzyme TPP1 [9]. CLN2 appears in previously healthy children aged 2–4 years, initially with tonic-clonic or partial seizures and delayed language acquisition. Progressive motor and cognitive decline occur about two years later, leading to the loss of voluntary movement, altered swallowing and speech, visual loss due to retinal atrophy (between 7 and 10 years), and death in mid-adolescence (10–15 years) [10,11]. Currently, therapy with enzyme replacement by infusion into the brain has been approved for the CLN2 disease [12,13].

Given the complexity of symptoms and their quick progression, CLN2 management requires support from families and a multidisciplinary team composed of pediatric neurologists, epileptologists, ophthalmologists, and physiotherapists. Management of CLN2 is aimed at optimizing the quality of life and reducing later-stage complications [14].

Cerliponase alfa is a recombinant human TPP1 enzyme used for replacement therapy administered through intracerebroventricular (ICV) infusion in patients with CLN2 [15]. In clinical studies, cerliponase alfa therapy proved to stabilize motor activity and language function loss in people affected by CLN2. An open-label, multi-center study assessed the efficacy and safety of cerliponase alfa infused in the brain through the intraventricular route over a period of at least 96 weeks in 23 children with CLN2 disease. The results showed a slowing decline in the motor–language score. Common adverse events included convulsions, pyrexia, vomiting, hypersensitivity reactions, and failure of the intraventricular device. In two patients, infections developed in the intraventricular device that was used to administer the infusion, which required antibiotic treatment and device replacement. Serious adverse events detected in this study included failure of the intraventricular device and device-related infections [16].

Cerliponase alfa was authorized in the European Union (EU) by the European Medicines Agency on 30 May 2017 for the treatment of CLN2 disease, also known as TPP1 deficiency. As reported in the ‘summary of product characteristics’, the drug must be administered only by a healthcare professional trained in intracerebroventricular infusion and in a healthcare setting at the recommended dose of 300 mg (lower doses in children less than 2 years old) administered once every other week [17]. Adverse reactions to cerliponase alfa were defined after exposure of 24 patients affected by CLN2 disease who received at least one dose of Brineura in clinical studies of up to 141 weeks. According to the pre-authorization clinical studies, the most common (>20%) adverse reactions detected were pyrexia, a low protein level in the cerebrospinal fluid, electrocardiogram alterations, vomiting, upper respiratory tract infections, and hypersensitivity. Treatment was never withdrawn due to these adverse reactions. It was authorized under “exceptional circumstances” because it was not possible to obtain complete drug information given the rarity of the disease. The above-reported safety profile of potential adverse reactions was confirmed in the last revision on 16 October 2023 (last updated product information) [17,18].

It has been suggested that we should explore ways to optimize the approaches we take by using real-world data to support regulatory decision-makers. In particular, for rare diseases, decisions based on randomized clinical trials may not always be practicable, while evidence associated with real-world data, despite its limitations, may play a fundamental role [19]. The safety profile of cerliponase alfa in patients with CLN2 is only partially known because knowledge of potential adverse reactions is based on information obtained with pre-authorization studies [15]. Moreover, no data exist on the post-marketing safety profile of cerliponase alfa treatment of CLN2.

The regulatory framework for pharmacovigilance does not distinguish between orphan and non-orphan drugs, but the surveillance of adverse reactions to orphan drugs can be more complicated. This is due, in particular, to the reduced sample size available of patients for an orphan drug indication. This factor can influence our knowledge of the safety profiles of orphan drugs either in the pre-authorization phase or in the post-marketing one. As such, the safety profile, in general, is relatively poorly known or it needs to be confirmed [20]. Here, with the aim of compiling clear information and improving our knowledge of the safety profile of cerliponase alfa in the treatment of neurolipofuscinosis, a descriptive analysis of real-world spontaneous reporting data of suspected adverse reactions to this drug was performed by searching the EudraVigilance database.

## 2. Results

ICSRs reporting suspected adverse reactions in EudraVigilance for the years from 2017 (June 1; date of first cerliponase-alfa-related ICSR) to 2023 (16 October) totaled 148. Individuals with SARs to cerliponase alfa were distributed according to their age as follows: 2 months–2 years 5.8%, 3–11 years 70.9%, 12–17 years 19.4%, and 18–64 years 3.9%. The most frequent adverse reactions were, in decreasing order, fever/pyrexia, device-related infection, positive cerebrospinal fluid culture, seizures/convulsions, vomiting, pleocytosis, irritability, ventriculitis, and respiratory disorders. Death was reported only in two cases (1.3%). All ICSRs related to cerliponase alfa reported neurolipofuscinosis as the therapeutic indication, the only one for which this drug is authorized.

In the cases reporting SARs to cerliponase alfa, the sex distribution showed a prevalence of females (58.1%) over males (41.9%). According to age, the sexes of patients in the cases reporting SARs were distributed as follows: 2 months–2 years 70% males and 30% females, 3–11 years 38% males and 62% females, 12–17 years 41.2% males and 58.8% females, and 18–64 years 33.3% males and 66.7% females. There was a prevalence of female ICSRs reporting fever/pyrexia, positivity of cerebrospinal fluid, pleocytosis, irritability, and respiratory disorders, while those of males were prevalent for device-related infections (Table 1).

For each ICSR (each case), more than one adverse reaction could be reported as occurring in the same patient. Consequently, number of adverse reactions was greater than the number of single cases. An analysis to establish the statistical significance of the differences in the occurrence of SARs according to the sex distribution was performed by using odds ratio calculation. The results showed that a statistically significant difference could be observed only in the greater number of cases with positivity of the cerebrospinal fluid culture in females (Table 2). Aggregation of the adverse reactions according to the System Organ Class (SOC) level showed that the groups of reactions most represented were “nervous system disorders”, followed by “general disorders and administration site conditions”, “infections and infestations”, “investigations”, and “respiratory, thoracic, and mediastinal disorders”. In Table 3, we list the classes of groups of adverse reactions signaled most frequently.

Based on the apparent gender prevalence in the occurrence of SARs related to cerliponase alfa use, we calculated the odds ratios for the ICRSs with the aim of defining the role of sex as a risk factor. The results, shown in Table 4, indicated that the slight prevalence of females in the reports of SARs was not statistically significant, either in patients showing more than one adverse reaction or in those with more than two adverse reactions to cerliponase alfa.

## 3. Discussion

As research in the field of rare pathologies grows, these diseases are now more often diagnosed, meaning they are becoming less infrequent and more commonly known, and there are still new rare pathologies to be discovered. However, there remain few studies investigating our pharmacovigilance concerning orphan drugs [21]. CLN2, also called Jansky–Bielschowsky disease, is a pathology caused by autosomal recessive mutations of the TPP1 gene, resulting in a deficiency in the lysosomal TPP1 [22,23]. Cerliponase alfa is the only approved therapy for CLN2, and, in this pathology, it produces a slowing decline in motor and language function in patients aged three or more years old, which occurs in most patients (83%) [24]. Pharmacovigilance systems include the adoption of activities for the analysis either of conventional or orphan drugs, and the post-marketing safety evaluation of drugs by researchers comprises analyses of the same aspects [25]. As detailed in this article, common adverse events to cerliponase alfa, according to data from pre-authorization studies, included convulsions, pyrexia, vomiting, hypersensitivity reactions, and failure of the intraventricular device function [16]. In our analysis of adverse reactions signaled following cerliponase alfa use, we found that pleocytosis occurred in 8.8% of the adverse reactions. This is notable since pleocytosis was not previously mentioned among the signs and symptoms reported with treatment using cerliponase alfa [13]. Regarding the possible occurrence of undesirable reactions after treatment with cerliponase alfa, research in laboratory animals showed that an antibody anti-cerliponase alfa could be induced in the cerebrospinal fluid and serum following intraventricular administration of the drug, without any influence on pharmacological activity or the occurrence of hypersensitivity reactions, as also shown in a Phase 1/2 clinical trial enrolling 24 subjects with CLN2 disease, who were treated intraventricularly with the enzyme [26]. Elsewhere, a multicenter, open-label study investigated the effects of intraventricular cerliponase alfa infused every 2 weeks in 24 children affected by CLN2 aged 3–16 years, at the dose of 300 mg for a period of about 96 weeks. The most common adverse reactions were convulsions, pyrexia, vomiting, hypersensitivity reactions, and failure of the intraventricular device, and in two cases, device-related infections required the use of antibiotics [16]. In another report, the frequency of adverse events was detected in a study investigating the effects of intraventricular cerliponase alfa infused every 2 weeks in 24 patients affected by CLN2 aged 3–16 years, at the dose of 300 mg for a period of around 48 weeks. All patients reported at least one adverse event and 23 of 24 patients experienced an adverse event considered to be related to cerliponase alfa administration. Study-drug-related events that occurred in at least 10% of the study population were included for analysis, with the following presentation: pyrexia 46% (11/24), hypersensitivity 33% (8/24), seizure 33% (8/24), epilepsy 17% (4/24), headache 13% (3/24), and vomiting 13% (3/24) [27].

An article outlining practical aspects of intracerebroventricular enzyme replacement therapy for CLN2 disease, based on 6 years of experience in treating 48 patients at the University Medical Center Hamburg-Eppendorf, showed that infection rates (also observed in our analysis as a secondary cause of an adverse reaction) and technical complications can be minimized and termination of treatment be avoided with strict adherence to best practice guidelines [28]. 

Intracerebral treatment with cerliponase alfa for CLN2 includes a time of infusion lasting more than four hours, to be replicated every 2 weeks for the whole life [29]. Consequently, device infection—referring to the presence of bacteria in the cerebrospinal liquid associated with fever, headache, and vomiting—can occur. Therapy for infection constitutes intravenous antibiotics and removal of the device [28].

The results of the present descriptive analysis generally confirmed the safety profile of cerliponase alfa in the treatment of CLN2 as it was established based on pre-authorization studies [21]. Compared to the pre-authorization studies, real-world data revealed the occurrence of pleocytosis, though no frequent electrocardiogram alterations were reported. Furthermore, when we considered the sex distribution of patients, an asymmetrical distribution of SARs was revealed.

Pleocytosis is defined as an increased cell count in the cerebrospinal fluid [30]. The clinical and prognostic significance of pleocytosis are not completely known and it is not clear whether it represents a negative prognostic sign [31]. Although its clinical significance is unclear, it is significant that this is the first time that it has been signaled in relation to the use of cerliponase alfa in the treatment of neurolipofuscinosis. Our data show that pleocytosis is slightly prevalent in females, and that it occurs in almost all cases associated with fever and headache.

When we considered the age distribution of SARs related to cerliponase alfa, our analysis revealed that SARs occurred prevalently in children aged 3–11 years (70.9%), followed by adolescents (19.4%). According to data referring to Western countries, where access to molecular diagnosis has become the standard of care over the last 10–15 years, epidemiology of neurolipofuscinosis indicates a global 0.6–0.7 per million inhabitants [15]. Since the European population, together with people of the United Kingdom, is estimated to be about 516 million, and the number of cases reporting SARs to cerliponase alfa is about 21 for each year in the period 2017–2023, it is possible to calculate approximately that the percentage of people with CLN2 reporting SARs is 47.8%. It indicates that adverse reactions to cerliponase alfa occur in one in two patients with CLN2.

SARs also occurred in a significant proportion of children aged from two months to two years (5.8%). This is an important finding since the safety and efficacy of cerliponase alfa in children aged less than 3 years are not yet well defined, as reported in the ‘summary of product characteristics’ for the drug authorization. Information on its use in two-year-old children is not sufficient, and there are no clinical data on its use in children aged less than two years (‘summary of product characteristics’; checked on 12 June 2023). From this point of view, the results of the present analysis indicate a need to pay further attention to the use of cerliponase alfa in very small children. Moreover, we have found that SARs occur, in general, more frequently in female patients, and this difference is particularly evident for fever/pyrexia, the positivity of cerebrospinal fluid, pleocytosis, irritability, and respiratory disorders.

Given that randomized controlled trials have limited generalizability and are insufficiently powered to detect rare adverse events [32], particularly in the case of orphan drugs, real-world data obtained through databases collecting spontaneous reports of adverse events can be used to confirm or unveil adverse reactions, as well as indicate the frequency of their occurrence.

## 4. Materials and Methods

**Figure 1:**
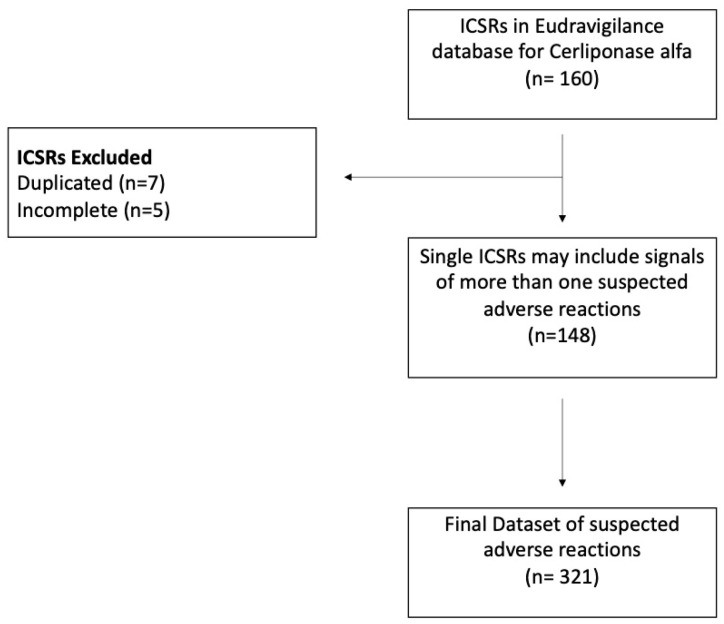
Flowchart of methodology. ICSRs (Individual Cases Safety Reports).

The EudraVigilance database collects spontaneous reported signals for adverse reactions to products licensed for the market. Suspected adverse reactions (SARs) to drugs are described as cases in individual case safety reports (ICSRs). ICSRs contain information on SARs as medical events that have been observed following the use of a medicine, but which have not necessarily been caused by the medicine. Each ICSR may include signals of more than one suspected adverse reaction, and for this reason, the number of cases can be different from the number of events [33]. The EudraVigilance data system contains all ICSRs reported to competent national authorities or marketing authorization holders. For our analysis, we reviewed ICSRs of SARs to cerliponase alfa from 1 June 2017 to 16 October 2023. The public version of the EudraVigilance database (https://www.ema.europa.eu/en/human-regulatory/research-development/pharmacovigilance/eudravigilance; checked on 16 October 2023) was used. ICSRs of SARs were selected on the basis of the Medical Dictionary for Regulatory Activities (MedDRA) (Introductory Guide MedDRA Version 26.0 March 2023. Available online: https://admin.meddra.org/sites/default/files/guidance/file/intguide_26_0_English.pdf; accessed and checked on 16 October 2023). MedDRA contains the standardized international medical terminology used by regulatory agencies and pharmaceutical companies. MedDRA terminology is also used to apply codes to cases with adverse events in clinical study safety reports and databases for pharmacovigilance [34]. Here, adverse reactions were described by using “Preferred Terms” (PTs) listed in MedDRA. A PT is a distinct descriptor (single medical concept) for an adverse symptom or an adverse sign. Two or more PTs with an overlapping clinical meaning were aggregated to avoid not-useful duplicate PTs with the same connotation (for example, the two PTs fever and pyrexia). MedDRA has a hierarchy of terms to describe adverse reactions. Adverse reactions are assembled into the System Organ Class (SOC) terms in the MedDRA hierarchy, such as musculoskeletal and connective tissue disorders, vascular disorders, etc. SOC is the highest level of the hierarchy and denotes the largest concept useful for retrieving data [35]. From each ICSR, data on the patient’s age and sex, the type of adverse event, source, and drugs taken concomitantly were extracted. All ICSRs were related to different patients, while duplicate and incomplete ICSRs were considered as missing data/not reported and excluded from the analysis. Criteria used to collect ICSRs related to cerliponase alfa from EudraVigilance were the following: SARs, complete for age and sex information, indication (according to medical terminology, this is intended here as “reason for use”), and concomitant drugs (Figure 1). A descriptive statistical analysis of data was carried out in the present study using the statistical software SPSS version 29.0 (Chicago Ill., USA). A stratification of signals by group for age and sex was used to reduce bias due to confounding effects caused by these variables. For patient age, the subgroups were 0–1 month, 2 months–2 years, 3–11 years, 12–17 years, 18–64 years, 65–85 years, and ≥85 years, and for patient sex, female and male formed the two subgroups. The ratio between the frequency with which for each case a suspected adverse reaction was signaled once or more than twice between males and females for the years 2017–2023 was calculated by using chi-square as the odds ratio (OR). The OR measures the degree of correlation between sex and the potential for more signals of SARs in single reports (ICSR) for males and females.

## 5. Conclusions

The limitations of the present research are those generally associated with findings discerned from databases containing real-world data of spontaneous reports of adverse drug reactions, with a point of strength being the number of reports and a weakness being the poor quality of individual signals [34]. Furthermore, this approach lacks a denominator, sometimes reducing the value of the conclusions drawn. Finally, it is important to remember that this kind of database contains single reports of adverse drug reactions considered as suspected, and consequently, we have to consider for any signaled adverse event a reasonable possibility but not a certainty that the drug caused it. However, databases regularly accumulating signals of SARs to drugs are considered fundamental resources in the field of pharmacovigilance since they furnish us with data from continuing and extensive surveillance in the real-world setting [35]. Analysis of real-world drug safety data is a method that may allow us to gain an early awareness of preventable adverse drug reactions and that may increase our knowledge about the safety profiles of drugs. The principal aim of the spontaneous reporting system is to allow us to quickly recognize new adverse drug reactions or discover differences in their severity or in gender distribution, and it has the advantage of involving a large portion of the population through low-cost investment [36].

In conclusion, the present descriptive analysis of post-marketing real-world data from a spontaneous signaling reporting system of adverse drug reactions against the orphan medicinal product cerliponase alfa confirms the safety profile established with clinical studies, but it suggests that clinicians should pay greater attention to pleocytosis as a possible predictive marker of prognostic value. Finally, although, at first glance, the results of the present study could be judged as bringing little scientific news, this is refutable, as specific problems remain around pharmacovigilance concerning orphan drugs and their use in rare diseases. Pre-authorization studies of orphan drugs are generally short-term studies conducted on a very limited population, and safety information about these drugs often originates from other therapeutic indications. In this context, any issue relating to safety is important to know, including this confirmation of the safety profile of a specific drug.

## Figures and Tables

**Table 1:** Frequency and sex distribution of suspected adverse reactions (SARs) to cerliponase alfa in the European Economic Area and the United Kingdom in the years 2017–2023.

Suspected Adverse Reaction	Males SARs	Males % of Total Number of SARs	Females SARs	Females % of Total Number of SARs	Total Cases with SARs	% of Total Number of SARs
Fever/Pyrexia	21	14.2	24	16.2	45	30.4
Device-related infection	16	10.8	11	7.4	27	18.2
Positive cerebrospinal fluid culture	4	2.7	17	11.5	21	14.2
Seizures/Convulsions	9	6.1	11	7.4	20	13.5
Vomiting	11	7.4	7	4.7	18	12.2
Pleocytosis	5	3.4	8	5.4	13	8.8
Irritability	3	2.0	6	4.0	9	6.1
Ventriculitis	4	2.7	2	1.3	6	4.0
Respiratory distress	0	0	3	2.0	3	2.0

Total number of cases = 148. Data are presented as number of cases or percentage of total number of cases. Only adverse reactions signaled at least two times were included in this table.

**Table 2:** Evaluation of odds ratios according to the sex distribution for single suspected adverse reactions (SARs) to cerliponase alfa in the European Economic Area (EEA) and the United Kingdom displayed in EudraVigilance for the years 2017–2023.

Suspected Adverse Reaction	Males SARs	Females SARs	OR (C.I.)	*p*-Value
Fever/Pyrexia	21	24	0.765625 (0.33–1.75)	0.263698
Device-related infection	16	11	2.1157024 (0.71–6.26)	0.088044
Positive cerebrospinal fluid culture	4	17	0.055363 (0.01–0.26)	0.000116
Seizures/Convulsions	9	11	0.669421 (0.19–2.33)	0.263891
Vomiting	11	7	2.469388 (0.65–9.43)	0.093076
Pleocytosis	5	8	0.390625 (0.08–1.90)	0.121820
Irritability	3	6	0.25 (0.03–1.77)	0.082829
Ventriculitis	4	2	4 (0.36–44.11)	0.128837
Respiratory distress	0	3	N/A	N/A

Total number of SARs = 321. OR = odds ratio; C.I. = confidence interval. N/A = not applicable. Only single adverse reactions signaled at least two times were included in this table.

**Table 3:** Suspected adverse reactions (SARs) to cerliponase alfa by reaction group signaled to EudraVigilance from the European Economic Area (EEA) and the United Kingdom in the years 2017–2023, according to the System Organ Class (SOC) level. Total number of SARs = 321.

Reaction Groups According to System Organ Class (SOC) Level	Number of Signaled SARs	% of Total Number of Signaled SARs
Nervous system disorders	77	24.0
General disorders and administration site conditions	67	20.9
Infections and infestations	42	13.1
Gastrointestinal disorders	28	8.7
Investigations	24	7.5
Respiratory, thoracic, and mediastinal disorders	20	6.2
Psychiatric disorders	14	4.4
Injury, poisoning, and procedural complications	11	3.4
Eye disorders	10	3.1
Skin and subcutaneous tissue disorders	9	2.8

Total number of SARs = 321. Data are presented as the number of SARs or the percentage of the total number of SARs. Only classes of adverse reactions signaled at least nine times were included in this table.

**Table 4:** Evaluation of odds ratios (OR) between male and female cases for individual case safety reports (ICSRs) signaling more than one or more than two suspected adverse reactions (SARs) to cerliponase alfa in the European Economic Area (EEA) and the United Kingdom, as displayed in EudraVigilance for the years 2017–2023. Total number of ICSRs = 148.

	Males (*n* = 62)	Females (*n* = 86)	OR 95% CI	*p*-Value
Number of cases with more than one adverse reaction	28/62	43/86	0.78 (0.41, 1.49)	0.224307
Number of cases with more than two adverse reactions	18/62	24/86	1.0568 (0.51, 2.18)	0.440458

Data are expressed as the number of ICSRs related to cerliponase alfa. *p* was considered significant when <0.05.

## Data Availability

The data analyzed and presented in this study are available on the public EudraVigilance data system.
